# Supplementary material for: Motion‐robust T2∗ quantification from low‐resolution gradient echo brain MRI with physics‐informed deep learning
Source: Magn Reson Med. 2025 Aug 22;95(1):346–62. doi: 10.1002/mrm.70050 (PMC12620166; doi:10.1002/mrm.70050)
Supplement: Supplementary file 1 — Data S1. Supporting Information Figure S1. Examples of reconstructed images (first echo) for networks trained conventionally (with original masks and no KeepCenter) and with the KeepCenter extension, compared to the fully sampled image. From top to bottom, the masks exclude three, five, and seven central k‐space lines. Green arrows indicate brain areas with more correctly recovered contrast in the KeepCenter reconstructions. Figure S2. Example exclusion masks estimated from inferior, middle, and superior slices, comparing the Even/Odd extension to an optimization of individual slices for two different motion patterns of the MoCo validation subject. For these acquisitions, the slice range based on the susceptibility gradient strength for the Even/Odd optimization is [10, 17]. These examples demonstrate a more stable‐line detection profile across the brain for the Even/Odd optimization compared to masks optimized for individual slices. Figure S3. Qualitative examples for applying PHIMO+ and the comparison methods to apparently motion‐free data. The resulting T2∗ maps (and, if available, exclusion masks) are compared for the original data without intentional motion, ORBA, SLD, PHIMO, PHIMO+, and HR/QR for two different subjects. Blue arrows indicate T2∗ quantification errors introduced by SLD. Figure S4. Acquisition excluded from the main analysis due to excessive motion in the k‐space center (10 s/nine lines). The T2∗ maps are compared for ORBA, SLD, PHIMO, PHIMO+, and HR/QR to the uncorrected acquisition (left) and the separate motion‐free acquisition (right). The respective exclusion masks are shown on the right. Green arrows indicate areas where PHIMO and PHIMO+, and to some extent HR/QR, clearly mitigate the extent of wave‐like motion artifacts, blue arrows indicate missing details across all methods. PHIMO and PHIMO+ overestimate the exclusion mask, likely due to excessive motion in the k‐space center, which challenges the reconstruction network even with the Kee [file MRM-95-346-s001.pdf]

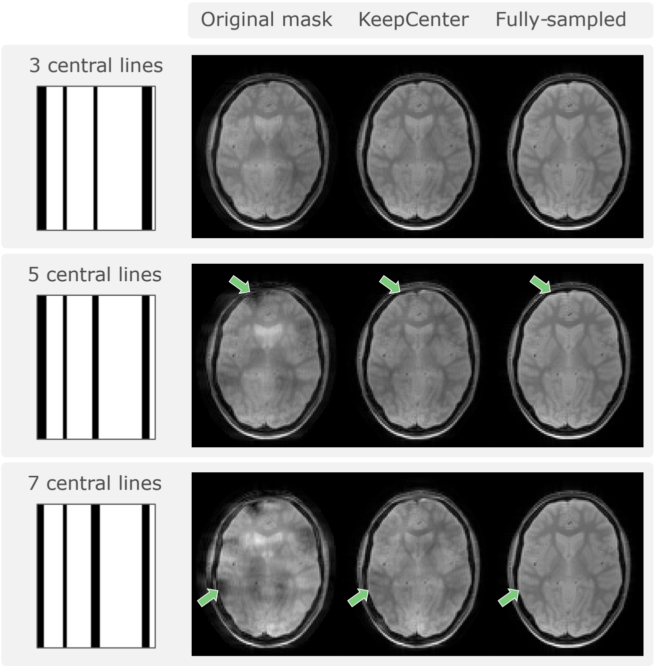

Figure S1: Examples of reconstructed images (first echo) for networks trained conventionally (with original masks and no *KeepCenter*) and with the *KeepCenter* extension, compared to the fully-sampled image. From top to bottom the masks exclude three, five and seven central k-space lines. Green arrows indicate brain areas with more correctly recovered contrast in the *KeepCenter* reconstructions.

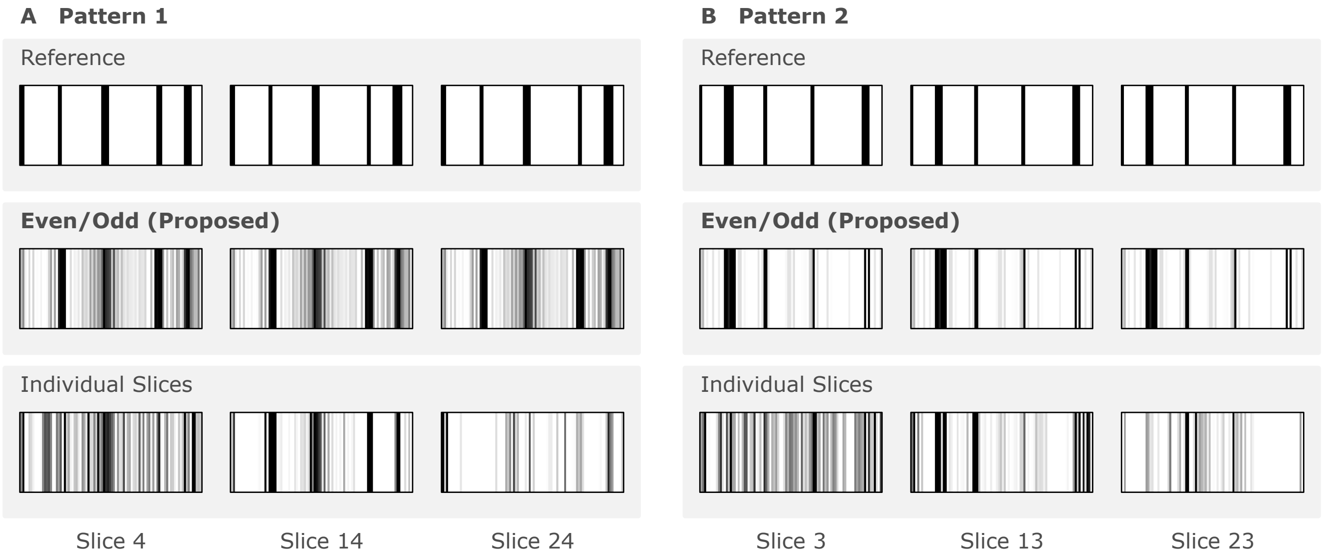

Figure S2: Example exclusion masks estimated from inferior, middle and superior slices, comparing the *Even/Odd* extension to an optimization of individual slices for two different motion patterns of the MoCo validation subject. For these acquisitions, the slice range based on the susceptibility gradient strength for the *Even/Odd* optimization is [10, 17]. These examples demonstrate a more stable line detection profile across the brain for the *Even/Odd* optimization compared to masks optimized for individual slices.

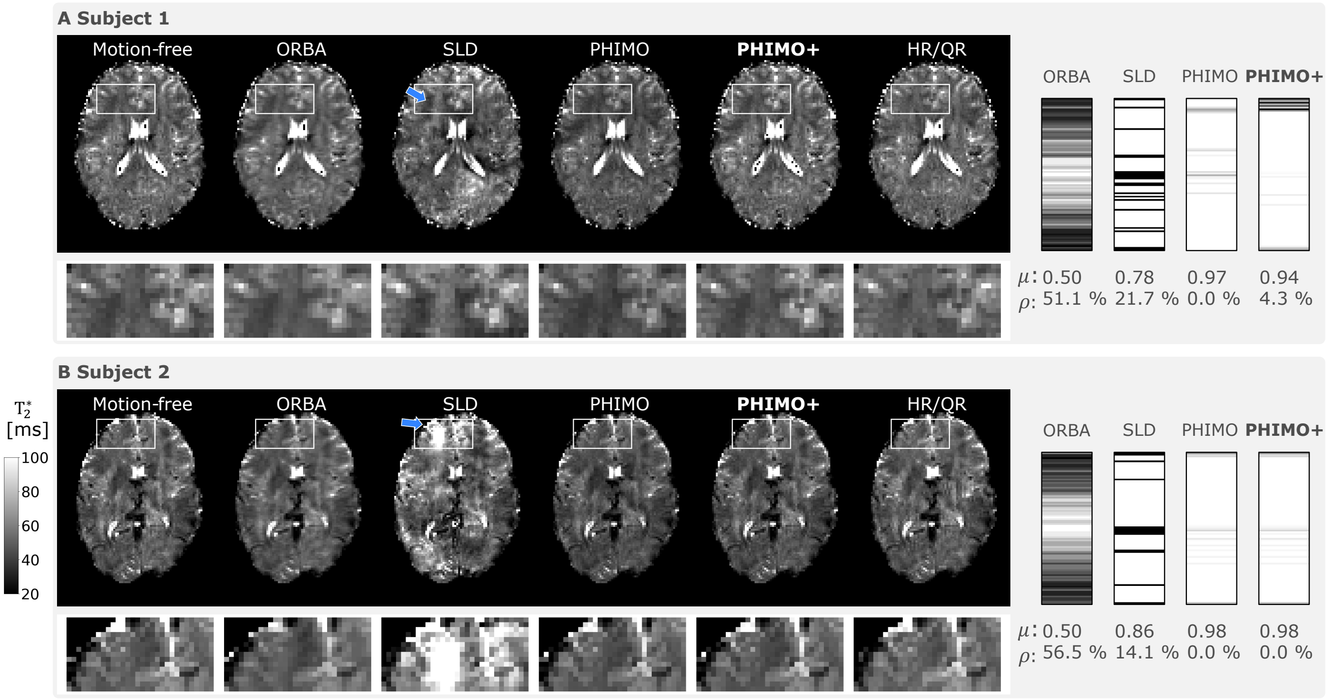

Figure S3: Qualitative examples for applying PHIMO+ and the comparison methods to apparently motion-free data. The resulting T_2^* maps (and, if available, exclusion masks) are compared for the original data without intentional motion, ORBA, SLD, PHIMO, PHIMO+ and HR/QR for two different subjects. Blue arrows indicate T_2^* quantification errors introduced by SLD. Average mask values (μ) and fraction of excluded lines (ρ) are provided below each mask.

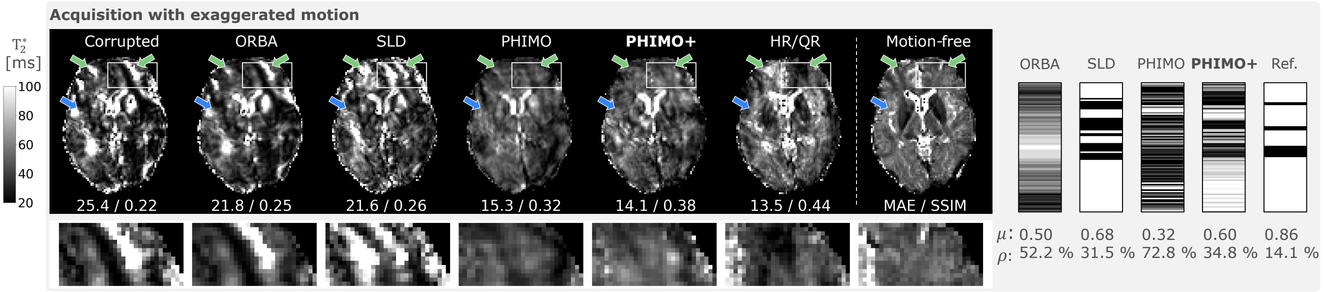

Figure S4: Acquisition excluded from the main analysis due to excessive motion in the k-space center (10 s / nine lines). The T_2^* maps are compared for ORBA, SLD, PHIMO, PHIMO+ and HR/QR to the uncorrected acquisition (left) and the separate motion-free acquisition (right). The respective exclusion masks are shown on the right. Green arrows indicate areas where PHIMO and PHIMO+ , and to some extent HR/QR, clearly mitigate the extent of wave-like motion artifacts, blue arrows indicate missing details across all methods. PHIMO and PHIMO+ overestimate the exclusion mask, likely due to excessive motion in the k-space center, which challenges the reconstruction network even with the *KeepCenter* extension. Note that the current standard, HR/QR, is also challenged by such an extreme motion case, which in clinical applications may ultimately require reacquisition. Average mask values (μ) and fraction of excluded lines (ρ) are provided below each mask.
